# Supplementary figures and images for: X-linked neonatal-onset epileptic encephalopathy associated with a gain-of-function variant p.R660T in GRIA3
Source: PLoS Genet. 2021 Jun 23;17(6):e1009608. doi: 10.1371/journal.pgen.1009608 (PMC8259962; doi:10.1371/journal.pgen.1009608)

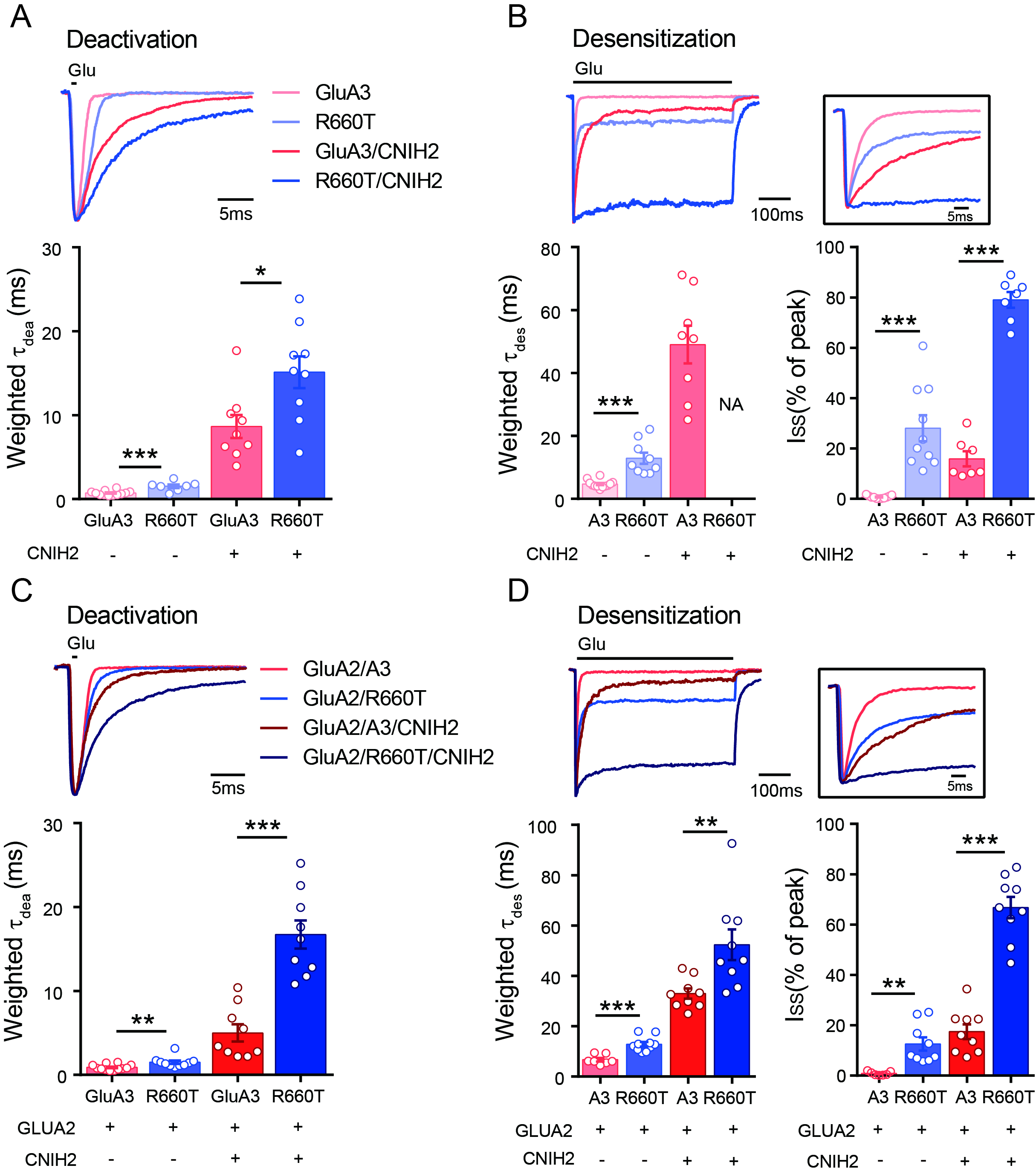

Supplement: S1 Fig — (A). Deactivation of GLUA3 and GLUA3_R660T in the presence of CNIH2. GLUA3 and GLUA3_R660T were the same as in Fig 1. GLUA3/CNIH2, 8.6 ± 1.4 ms, n = 9; R660T/CNIH2, 15.1 ± 1.9 ms, n = 9; *p = 0.0136. (B) Up, the sample traces for desensitization of GLUA2/A3 and GLUA2/A3_R660T in the presence of CNIH2. Low left, statistics of weighted τdes. GLUA3/CNIH2, 49.0 ± 6.0 ms, n = 8; R660T/CNIH2, no clear desensitization and τdes value cannot be calculated, n = 9. Low right, statistics of steady state currents. GLUA3/CNIH2, 15.9 ± 3.0%, n = 7; R660T/CNIH2, 79.1 ± 3.2%, n = 7; ***p < 0.001. (C) Deactivation of GLUA2/A3 and GLUA2/A3_R660T in the presence of CNIH2. GLUA2/A3 and GLUA2/A3_R660T were the same as in Fig 2. GLUA2/A3/CNIH2, 5.0 ± 1.0 ms, n = 9; GLUA2/A3_R660T/CNIH2, 16.7 ± 1.7 ms, n = 9; ***p < 0.001. (D) Up, the sample traces for desensitization of GLUA2/A3/CNIH2 and GLUA2/A3_R660T/CNIH2. Low left, statistics of weighted τdes. GLUA2/A3/CNIH2, 33.0 ± 2.0 ms, n = 9; GLUA2/A3_R660T/CNIH2, 52.4 ± 6.1 ms, n = 9; **p = 0.008. Low right, statistics of steady state currents. GLUA2/A3/CNIH2, 17.5 ± 2.9%, n = 9; GLUA2/A3_R660T/CNIH2, 66.8 ± 4.3%, n = 9; ***p < 0.001. Data are presented as mean ± SEM. Unpaired t-test was used for data analysis. (TIF) [file pgen.1009608.s001.tif]
