# Supplementary material for: X-linked neonatal-onset epileptic encephalopathy associated with a gain-of-function variant p.R660T in GRIA3
Source: PLoS Genet. 2021 Jun 23;17(6):e1009608. doi: 10.1371/journal.pgen.1009608 (PMC8259962; doi:10.1371/journal.pgen.1009608)
Supplement: Attachment — Submitted filename: Point-to-point answers to Reviewers Comments.docx [file pgen.1009608.s002.docx]

Reviewer's Responses to Questions

**Comments to the Authors:
Please note here if the review is uploaded as an attachment.**

Reviewer #1: In the Manuscript entitled "X-linked neonatal-onset epileptic encephalopathy associated with a gain-of-function variant p.R660T in GRIA3", Sun J. et al. identified a pathogenic missense variant in GRIA3 gene in a female patient with severe epilepsy and global developmental delay. Through electrophysiological analysis the authors found this mutant makes strengthened glutamate receptors, which indicates that the variant causes epileptic encephalopathy and global developmental delay by enhancing glutamate signaling in brain. This is a very interesting story and set a good example for the combination for clinical and basic research. Please address the following questions before being considered for publication.

This reviewer praised our study ***a very interesting story and set a good example for the combination for clinical and basic research*.** We deeply appreciate his/her nice comments.

1. Other than the little girl mentioned in this study, how many more patients are found carrying this A3 mutation?

This is the only patient carrying mutation on the R660 site thus far. There are other mutations on GRIA3 published that are mostly found in male patients.

2. After whole genome sequencing, how many site mutation(s) or SNPs are discovered in the little girl? If more mutations are found, what roles do those mutations play in epilepsy?

This is a critical point. As outlined in the Methods section, to identify candidate variants of potential clinical interest, variants were filtered and considered based on multiple factors including population allele frequency (<1% for SNVs and indels, <10% for CNVs), variant consequence, evolutionary conservation, occurrence in a gene with a well-established gene-disease relationship, occurrence in a gene whose disease association overlaps with the patient’s reported phenotype, and inheritance mode, as appropriate. Clinical interpretation was performed on variants and CNVs of interest in accordance with the American College of Medical Genetics and Genomics guidelines.

A list of genes was generated by searching the Online Mendelian Inheritance In Man (OMIM) database for genes that have been associated with key elements of the phenotype. This directed query yielded a list of approximately 8000 genes, which was used to prioritize resulting variants from the family-based analysis and to guide secondary analyses of only the patient’s genome.

All variants related to the patient’s phenotype were ruled out based on a synthesis of available information. No additional variants of clinical interest in genes associated with epilepsy or other aspects of the patient’s phenotype were identified.

We add this information in revised manuscript (Page 5, Line7-8).

3. In figures 5 and 6, what roles does endogenous GluA3 play?

In this study we have not examined the role of endogenous GluA3. We choose CGNs and hippocampal CA1 neurons as the model systems to understand the GLUA3 mutation on synaptic neurotransmission. These two types of neurons have very limited endogenous GluA3 and perhaps this is the reason that the overexpressed GLUA3 and R660T mutation show very dramatic effects on the excitatory synaptic currents.

Based on literature, GluA3 may contribute a major role (over GluA1) in synaptic AMPARs in cerebellar Purkinje neurons (Gutierrez-Castellanos N., et al. Neuron 2017). In cortex, the expression level (Schwenk J. et al., Neuron 2014) suggests that GluA3 might play a role equal to GluA1, yet this has not been functionally analyzed. Considered the mutated GLUA3-R660T has such a dramatic consequence, we believe that endogenous GluA3 might play more roles in some brain regions than that has been revealed in hippocampus (Lu W, et al. Neuron 2009).

Reviewer #2: In this study the authors identified a female individual exhibiting epileptic encephalopathy and harboring a missense mutation in GRIA3 gene resulting in a GluA3 R660T protein variant. Expression of the R660T GluA3 mutant alone in HEK cells resulted in a marked slowing of GluA3 homomer deactivation and desensitization kinetics and produced a large increase in steady state current of the channel. This mutation produced similar effects in GluA2/A3 heteromeric channels in HEK cells. The effects of this mutation were also present with GluA2 homomers and GluA2/A3 heteromers associated with y-2 and CNIH2 auxiliary subunits. Additionally, overexpression of GluA3 R660T in cerebellar granule neurons produced slower mEPSC decay kinetics when compared to overexpression of wild-type GluA3. GluA3 R660T overexpression in CA1 pyramidal neurons was also shown to increase AMPAR-eEPSC charge transfer and slow AMPAR-eEPSC and mEPSC decay kinetics compared to wild-type GluA3 overexpression.

Overall this is a very interesting and carefully executed study that clearly describes the nature of the glutamatergic synapse perturbation that likely contributes to this individual’s epileptic encephalopathy. While a molecular replacement approach where GluA3 is removed and replaced with GluA3 R660T might be preferable to the overexpression expression experiments performed, I do not regard this as a major limitation of the study. From the work the authors’ have already done it is very clear what this mutation is doing to AMPAR function. I would have no problem with the manuscript being published in its current form.

We are delighted that this reviewer also praised our study ***a very interesting and carefully executed study.*** Thanks!

Minor issues:
1) Were mutations found in any other genes associated with epilepsy in this individual?

This is similar to the 2nd question raised by the first reviewer. Please refer to our answer to that question. A short answer is that we do not find any other mutations related to the patient’s phenotype.

2) In the first paragraph of the introduction “Though GLUA3 alone can form Ca2+ permeable homomeric receptors, in the brain it mainly forms heteromeric receptors with GLUA2” needs a reference.

We added a reference (Page 3, line 8-9). Thanks.
